# Acute Recreational Cannabis-Induced Hypersensitivity Pneumonitis: A Case Report

**DOI:** 10.7759/cureus.37312

**Published:** 2023-04-08

**Authors:** Nicholas D Luke, Baris Vefali, Priscilla Chow, Richard Miller

**Affiliations:** 1 Emergency Department, St. Barnabas Hospital Health System, The Bronx, USA; 2 Internal Medicine, Saint Michael's Medical Center, Newark, USA; 3 Pulmonology and Critical Care, Saint Michael's Medical Center, Newark, USA

**Keywords:** chest x-ray, steroid treatment, lung opacity, pulmonology, cannabis use

## Abstract

Hypersensitivity pneumonitis (HP) is a lung disease in which foreign matter is inhaled and exposed to lung parenchymal and interstitial tissue. Such matter may include pollen, molds, chemicals, and smoke. HP leads to widespread inflammation and even fibrosis in chronic forms; the main route of treatment usually involves corticosteroids and antifibrotics as needed. We describe a patient case in which HP was diagnosed after using recreational marijuana, and her chest x-ray had a complete resolution after one day of a corticosteroid regimen. As recreational marijuana use increases, clinicians need to keep HP on the differential diagnosis in patients that frequently utilize recreational marijuana obtained through illicit business.

## Introduction

Hypersensitivity pneumonitis occurs with lung exposure to foreign media that may be organic or inorganic. The presentation of hypersensitivity pneumonitis (HP) can be highly variable, depending on the type of media the patient has been exposed to, including chemicals, mold, and bacteria. HP can be divided into acute, subacute, and chronic. The acute form may present with diffuse alveolar, interstitial, and bronchiolar inflammation, whereas the subacute and chronic forms may present with noncaseating granulation tissue within the interstitium [1]. One particular foreign organic media that can induce HP is cannabis use. Tashkin examined studies showing that habitual or daily use of cannabis can lead to acute and/or chronic respiratory diseases, such as infection, bronchitis, pneumonia, and so on. The frequent use of cannabis can also impact airway function and alter the cellular structure. Histopathological evidence in cannabis users was noted. It included airway injuries in the form of vascular hyperplasia, submucosal edema, inflammatory cell infiltrates, and hyperplasia of surface mucus-secreting goblet cells [2]. The management of HP is typically based on radiological imaging. However, there is no "gold standard" test. Once the diagnosis is clinched, treatment usually involves immunosuppressant drugs, such as steroids, and patient education on reducing exposure to the insulting antigen. Other immunosuppressant drugs that have shown efficacy include pirfenidone, an antifibrotic, and nintedanib, a kinase inhibitor that binds to fibroblast growth factor receptors [3]. This case report presents a patient with cannabis-induced hypersensitivity pneumonitis with resolution after one day of steroid therapy.

## Case presentation

A 48-year-old female initially presented with chest and epigastric pain for three days. She has a history of hypertension, hyperlipidemia, diabetic neuropathy, and coronary artery disease status post remote stent placement. The review of systems was also significant for a dry cough, shortness of breath, night sweats, and swelling of the abdominal wall and lower extremities for the last two days. The patient endorsed smoking marijuana several times weekly (for the last few months, as per the patient) but denied using tobacco products. She received complete coronavirus disease 2019 (COVID-19) vaccination at that time. Her vital signs and body mass index were unremarkable. The physical examination was pertinent for crackles in the bilateral lower lung fields and tenderness in the epigastric, right, and left upper quadrants. Her COVID-19 antigen, polymerase chain reaction, rapid flu test, and flu A and B results were all negative.

**Figure 1:**
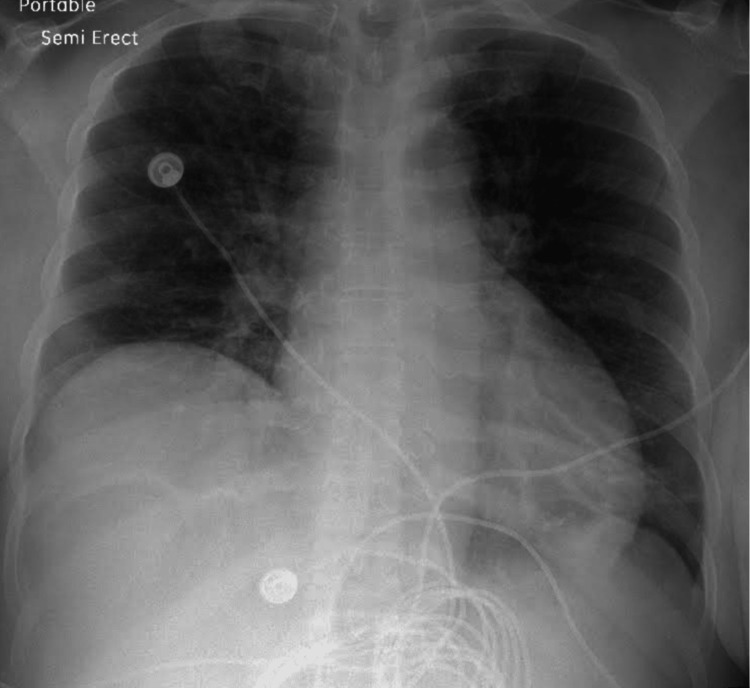
The initial chest x-ray depicting right upper lobar and diffuse left lung airspace opacities, possibly suggesting atypical pneumonia.

The troponin and brain natriuretic peptide results were unremarkable. However, her D-dimer was moderately elevated at 1171 mg/L FEU, and the albumin level was 2.4 g/dL on admission. The patient's liver function tests showed aspartate transaminase, alanine transaminase, and alkaline phosphatase levels of 119 U/L, 205 U/L, and 130 U/L, respectively. An abdominal ultrasound showed gallbladder wall thickening and pericholecystic fluid. A computed tomography scan of the lumbar spine with contrast showed atherosclerotic calcifications. An initial chest x-ray depicted right upper lobar and diffuse left lung airspace opacities, with no signs of effusion or pneumothorax (Figure 1). A computed angiogram of the chest showed no evidence of pulmonary embolism. However, bilateral intralobular septal thickening was scattered throughout all lung fields, along with ground glass opacities suspicious of pulmonary edema or an inflammatory process.

**Figure 2:**
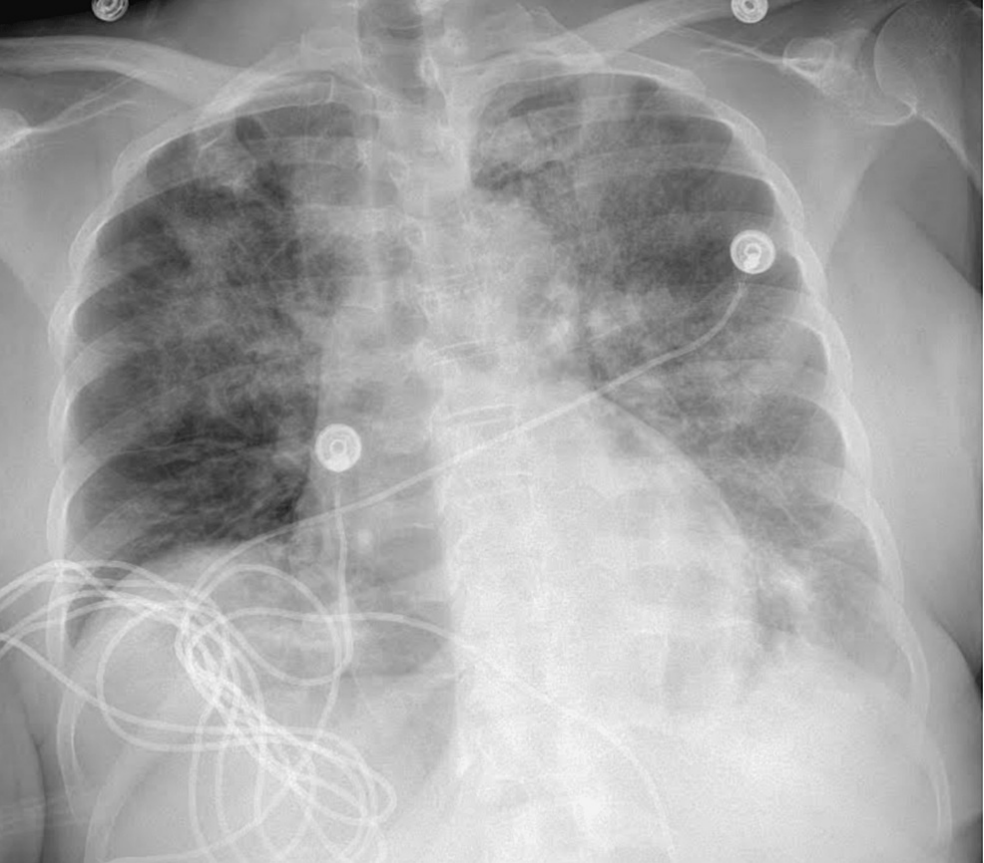
X-ray taken one day after starting a steroid regimen. Although bilateral parenchymal opacities are still present, there is a significant improvement compared to Figure 1.

A negative hepatobiliary iminodiacetic acid (HIDA) scan ruled out acute cholecystitis. The patient was admitted, and the plan was to order hepatitis and autoimmune workup while starting steroid therapy. Another x-ray was taken one day after starting the steroid regimen, and it showed a significant improvement in clearing the bilateral parenchymal opacities compared to the day before (Figure 2). The patient was cleared by pulmonology and discharged the next day.

## Discussion

HP is an airway sensitivity disease that arises from repeated exposure to inhaled antigens that insult the lung parenchymal and interstitial tissues. The epidemiology may vary since HP is challenging to diagnose due to variations in severity and the type of antigen exposed. Pérez et al. examined the epidemiology via an algorithm and concluded that the disease incidence and prevalence tended to be more predominant in females and increased further with age in patients older than 65 years of age. Pérez et al. also noted that more than half of the cases they studied were chronic and that the mortality rate increased if the HP presented with pulmonary fibrosis [4]. Cases with underlying pulmonary fibrosis should be managed promptly with antifibrotics and immunosuppressants, such as steroids.

Recreational cannabis use may pose a higher risk of HP because it is more likely to have impurities. Such impurities can include chemical additives and human contamination leading to mold and microbial growth. In one example, a patient inhaled a large concentration of tetrahydrocannabinol via dabbing, leading to a diagnosis of HP. Dabbing can also have impurities, especially concentrations of unpurged butane vapors, benzene, and methacrolein. These impurities can be carcinogenic and cause lung interstitial and parenchymal tissue dysplasia. While the exact pathophysiologic mechanism is unknown, Haddad states that HP may result from a direct inhalation injury or a maladaptive immunological response by the body to the chemical impurities. In a study by Haddad et al., it is possible that the concentrated butane vapors led to an insult within the lungs upon inhalation and led to HP [5]. Our presenting patient may have had a similar underlying pathophysiological mechanism of injury, but the treatment and management were the same.

Since HP has many variations in its presentation and a plethora of underlying triggers, it is difficult to differentiate it from other interstitial lung diseases. For example, idiopathic pulmonary fibrosis patients may appear to have chronic HP [6]. This is due to chronic HP having features of fibrosis as well. Thus, pulmonologists may misdiagnose patients with chronic HP as idiopathic pulmonary fibrosis instead [6]. To combat this, clinicians should closely examine environmental factors to rule out chronic HP. If an underlying environmental factor is suspected, the patient should be educated on removing the trigger promptly and preventing further disease progression.

## Conclusions

As the popularity of recreational cannabis increases, there is more potential for cases of HP to present themselves. Recreational cannabis may more likely contain impurities when obtained through illicit street business since it is less regulated than cannabis sold in dispensaries. Exposure to such impurities may insult the lungs, as is the case with our presenting patient. After taking steroids for one day, our patient had complete resolution of the chest x-ray, which is a remarkable speed of the healing process. We recommend clinicians keep a high index of suspicion for HP in patients who smoke cannabis frequently and habitually; treatment should involve immunosuppressants and antifibrotics if needed in patients with more chronic disease processes.
